# A Role for Malignant Brain Tumor Domain-Containing Protein 1 in Human Endometrial Stromal Cell Decidualization

**DOI:** 10.3389/fcell.2020.00745

**Published:** 2020-08-11

**Authors:** Sangappa B. Chadchan, Vineet K. Maurya, Gwendalyn L. Krekeler, Emily S. Jungheim, Ramakrishna Kommagani

**Affiliations:** ^1^Department of Obstetrics and Gynecology, Washington University School of Medicine, St. Louis, MO, United States; ^2^Center for Reproductive Health Sciences, Washington University School of Medicine, St. Louis, MO, United States; ^3^Department of Obstetrics and Gynecology, Fienberg School of Medicine, Chicago, IL, United States

**Keywords:** decidualization, malignant brain tumor domain-containing protein 1 (MBTD1), progesterone, endometrium, stromal cells, embryo implantation

## Abstract

Up to 30% of women experience early miscarriage due to impaired decidualization. For implantation to occur, the uterine endometrial stromal fibroblast-like cells must differentiate into decidual cells, but the genes required for decidualization have not been fully defined. Here, we show that Malignant Brain Tumor Domain-containing Protein 1 (MBTD1), a member of the polycomb group protein family, is critical for human endometrial stromal cell (HESC) decidualization. MBTD1 predominantly localized to HESCs during the secretory phase and the levels were significantly elevated during *in vitro* decidualization of both immortalized and primary HESCs. Importantly, siRNA-mediated *MBTD1* knockdown significantly impaired *in vitro* decidualization of both immortalized and primary HESCs, as evidenced by reduced expression of the decidualization markers PRL and *IGFBP1*. Further, knockdown of *MBTD1* reduced cell proliferation and resulted in G2/M cell cycle arrest in decidualizing HESCs. Although progesterone signaling is required for decidualization, MBTD1 expression was not affected by progesterone signaling; however, *MBTD1* knockdown significantly reduced expression of the progesterone target genes *WNT4*, *FOXOA1*, and *GREB1*. Collectively, our data suggest that MBTD1 contributes to *in vitro* decidualization of HESCs by sustaining progesterone signaling. This work could have implications for designing diagnostic and therapeutic tools for recurrent pregnancy loss.

## Introduction

Approximately 30% of women experience early miscarriage due to defective decidualization. Many of these miscarriages likely occur because the uterine endometrial stromal cells fail to transform from fibroblastic stromal cells into epithelioid-like secretory decidual cells (Salker et al., 2010; Teklenburg et al., 2010; Koot et al., 2012). To develop better diagnostic tools and treatment options for women experiencing early miscarriages, we must fully define the mechanisms and proteins required for decidualization.

Two key players in decidualization are the steroid hormones estrogen and progesterone. The primary role of estrogen is to prepare the uterine epithelia for embryo implantation. When progesterone concentration increases, estrogen action is inhibited in uterine epithelia, and endometrial stromal cell decidualization initiates. Progesterone acts by binding progesterone receptors and associated coregulators and subsequently activating expression of genes (Rubel et al., 2010; Lonard and O’Malley, 2012; Hantak et al., 2014; Wang et al., 2017) such as *FOXO1A*, *WNT4*, *GATA2*, and *GREB1*. However, it is likely that many genes important for decidualization have not been identified.

Bian et al. showed that polycomb repressive complex 1 (PRC1) controls decidualization (Bian et al., 2016). Polycomb group (PcG) proteins play important roles in development through their ability to modify histones and thereby control gene expression (Luo et al., 2013). Two PcG protein complexes, PRC1 and polycomb repressive complex 2 (PRC2), regulate chromatin remodeling and affect gene regulation (Levine et al., 2002). The PcG protein malignant brain tumor domain-containing protein 1 (MBTD1) regulates gene expression by controlling the chromatin remodeling process. MBTD1 is also a part of the histone acetyltransferase TIP60/NuA4 complex, which participates in DNA double-strand breaks repair through homologous recombination (Jacquet et al., 2016). Interestingly, acting as histone modifiers, the PcG proteins regulate gene transcription through methyltransferase activity (Mills, 2010) and by modulating histone acetyltransferase activity of the NuA4/TIP60 complex (Jacquet et al., 2016). Furthermore, histone modifications have been shown to play an important role in decidualization (Liu et al., 2019).

The MBTD1–CXorf67 fusion proteins have been observed in cases of low-grade endometrial stromal sarcoma (Dewaele et al., 2014). Further, analysis of publicly available GEO datasets revealed distinct MBTD1 expression in endometrial tissue in both physiological and pathological conditions, suggesting a possible role for MBTD1 in endometrial function. Here, we demonstrate that MBTD1 is upregulated during decidualization and plays an important role in progesterone-driven human and mouse endometrial stromal cell decidualization.

## Materials and Methods

### Ethical Approval for the Collection of Endometrial Tissue

Endometrial tissue from women with regular menstrual cycles were obtained under a protocol (IRB ID #201612127) approved by the Washington University in St. Louis Institutional Review Board and the guidelines of the Declaration of Helsinki (Michalski et al., 2018). All subjects were recruited through the Washington University online classified section and local newspaper advertisements. Eligible participants signed an Informed Consent and Authorization form.

### Endometrial Stromal Cell Isolation

Primary human endometrial stromal cells (HESCs) were obtained from patient donors. Patients provided written informed consent, and the research was approved by the Washington University in St. Louis Institutional Review Board (IRB ID #201612127) as described previously (Michalski et al., 2018). Additionally, all work involving human subjects followed the guidelines of the World Medical Association Declaration of Helsinki. Endometrial biopsies from healthy women of reproductive age were obtained during the proliferative phase (days 9 to 12) of the menstrual cycle, and endometrial cells were isolated as described previously (Camden et al., 2017; Michalski et al., 2018). Briefly, endometrial biopsies were minced using sterile scissors and then digested in DMEM/F12 media containing 2.5 mg/ml collagenase (Sigma-Aldrich, Saint Louis, MO, United States) and 0.5 mg/ml DNase I (Sigma-Aldrich, St. Louis, MO, United States) for 1.5 h at 37°C. Subsequently, detached cells were centrifuged and collected. The cells were then layered over a Ficoll-Paque reagent layer (GE Healthcare Biosciences, Pittsburgh, PA, United States) to remove lymphocytes. The top layer, containing the HESC fraction, was collected and filtered through a 40-μm nylon cell strainer (BD Biosciences, Franklin Lakes, NJ, United States). The filtrate containing HESCs was further resuspended in DMEM/F12 media containing 10% fetal bovine serum (FBS), 100 U/ml penicillin, and 0.1 mg/ml streptomycin and cultured in tissue culture flasks. For each experiment, the total number of a minimum of three HESC lines were used. Representative data are from one patient sample experiment performed with three technical replicates.

### Cell Culture

Primary HESCs were maintained in DMEM/F12 media supplemented with 10% FBS, 100 U/ml penicillin, and 0.1 mg/ml streptomycin at 37°C and 5.0% CO_2_ concentration under humid conditions. Experiments were performed using no more than four passages. The telomerase-transformed human endometrial stromal cells (T-HESCs) were purchased from ATCC (ATCC CRL4003) and maintained in phenol-red free DMEM/F12 medium with 3.1 g/L glucose and 1 mM sodium pyruvate supplemented with 10% charcoal/dextran-treated FBS, 1% ITS (insulin, transferrin, sodium selenite + Premix, 1.5 g/L sodium bicarbonate, and 500 ng/ml puromycin), 100 U/ml penicillin, and 0.1 mg/ml streptomycin. The media was replaced every other day.

### siRNA Transfection and *in vitro* Decidualization

HESCs were plated in 6-well cell culture plates and treated in triplicate with Lipofectamine 2000 reagent (Invitrogen Corporation, Carlsbad, CA, United States) and 60 pmol of the following siRNAs: non-targeting siRNA/control siRNA (D-001810-10-05) or siRNAs targeting *MBTD1* (L-020603-00-005) (GE Healthcare Dharmacon Inc., Lafayette, CO, United States) as described previously (Camden et al., 2017). After 48 h, cells were treated with decidualization media containing 100 nM estradiol (cat. no. E1024, Sigma-Aldrich), 10 μM medroxyprogesterone 17-acetate (MPA) (cat. no. M1629, Sigma-Aldrich), and 50 μM N6, 2′-O-dibutyryladenosine 3′,5′-cyclic monophosphate sodium salt (cat. no. D0260, Sigma-Aldrich) in 1 × Opti-MEM reduced-serum media containing 2% charcoal-stripped FBS (referred to as EPC). The decidualization medium was changed every 48 h until day 3 or day 6, when the cells were harvested for RNA isolation with a total RNA isolation kit (Invitrogen/Life Technologies, Grand Island, NY, United States).

For MPA time course experiments, HESCs were plated in six-well cell culture plates and, at 80–90% confluence, were treated with 1 μM MPA in 1 × Opti-MEM reduced-serum media containing 2% charcoal-stripped FBS. Cells were harvested for RNA isolation using a total RNA isolation kit (Invitrogen/Life Technologies) or for protein collection using RIPA buffer (cat. no. 9806, Cell Signaling Technology) containing 20 mM Tris–HCl (pH 7.5), 150 mM NaCl, 1 mM Na_2_EDTA, 1 mM EGTA, 1% NP-40, 1% sodium deoxycholate, 2.5 mM sodium pyrophosphate, 1 mM beta-glycerophosphate, 1 mM Na_3_VO_4_, 1 μg/ml leupeptin, and 1 mM PMSF.

### Cell Proliferation Assay

Cell proliferation was determined by performing the MTT assay (Promega, Madison, WI, United States) according to the manufacturer’s instructions. Briefly, HES cells were transfected with non-targeting/control siRNA (D-001810-10-05), *MBTD1* siRNA (L-020603-00-005), and *PGR* siRNA (L-006763-00-0005) (GE Healthcare Dharmacon Inc., Lafayette, CO, United States) and co-transfected with both *MBTD1* and *PGR* siRNAs with the aid of Lipofectamine 2000 reagent (Invitrogen Corporation, Carlsbad, CA, United States). Then, 48 h post-transfection, 1 × 10^3^ HESCs were plated per well of a 96-well plate (in triplicate). After the attachment, cells were treated with decidualization (EPC) media. The relative proliferation rate was evaluated with the MTT proliferation kit at 0, 24, and 48 h. The experiments were performed on three independent HESCs lines with three technical replicates in each.

### Flow Cytometry

Forty-eight hours following siRNA transfection with control siRNA or *MBTD1* siRNA alone or *PGR* siRNA alone or both *MBTD1* and *PGR* siRNAs together using Lipofectamine 2000 transfection agent (Invitrogen Corporation, Carlsbad, CA, United States), HESCs were trypsinized and counted. For flow cytometry analysis, 2 × 10^5^ cells were plated per well of six-well plates and then were treated with decidualization (EPC) media. After cells were cultured in EPC media for 48 h, cells were trypsinized, washed with phosphate-buffered saline (PBS), fixed in 70% chilled ethanol, and stained with 50 μg/ml propidium iodide (Sigma-Aldrich, St. Louis, MO, United States) containing 50 μg/ml RNase. Cell cycle stage analysis was performed by flow cytometry (FACS Canto II) and FACS Diva software (ver. 8.0; BD Biosciences, Franklin Lakes, NJ, United States). Each experiment was performed in technical duplicates and repeated in three independent HESC lines isolated from three subjects.

### Prolactin ELISA

After 48 h of transfection with control or *MBTD1* siRNA, cells were treated with decidualization media, and media was changed every 48 h until day 6. The cell culture supernatant was collected at day 0 and 6 and stored at −80°C. According to the manufacturer’s instructions, the Prolactin ELISA (cat. no. EHIAPRL, Invitrogen) was performed in cell culture media. Briefly, 50 μl of media was used to quantify the secreted Prolactin protein, and concentration of Prolactin was calculated from the standard curve. Each experiment was performed in triplicate and repeated in three independent HESC lines isolated from three subjects.

### qRT-PCR

Cells or tissues were lysed in lysis buffer, and total RNA was isolated with the Purelink RNA mini kit (Invitrogen) according to the manufacturer’s instructions. RNA was quantified with a Nano-Drop 2000 (Thermo Scientific, Waltham, MA, United States). RNA (1 μg) was reverse transcribed with the High-Capacity cDNA Reverse Transcription Kit (Thermo Scientific). The amplified cDNA was diluted to 10 ng/μl, and qRT-PCR was performed with primers specified in Supplementary Table 1 and Fast Taqman 2 × mastermix (Applied Biosystems/Life Technologies, Grand Island, NY, United States) on a 7500 Fast Real-time PCR system (Applied Biosystems/Life Technologies). The delta–delta cycle threshold method was used to normalize expression to the reference gene 18S (Kommagani et al., 2013, 2016; Camden et al., 2017).

### SDS-PAGE and Western Blotting

HESCs were homogenized in RIPA lysis buffer (cat. no. 9806, Cell Signaling Technology) containing 20 mM Tris–HCl (pH 7.5), 150 mM NaCl, 1 mM Na_2_EDTA, 1 mM EGTA, 1% NP-40, 1% sodium deoxycholate, 2.5 mM sodium pyrophosphate, 1 mM beta-glycerophosphate, 1 mM Na_3_VO_4_, 1 μg/ml leupeptin, and 1 mM PMSF and centrifuged at 14,000 × *g* for 15 min at 4°C to collect the supernatant containing total protein lysates. Protein concentration was quantified using the BCA Protein Assay kit (cat no. 23227, Thermo Scientific) according to the manufacturer’s instructions. HESC lysate sample, containing 40 μg of protein, was loaded on a 4–15% SDS-polyacrylamide gel, separated with 1 × Tris–Glycine running buffer, and transferred to PVDF membranes using a wet electro-blotting system (Bio-Rad, United States), according to the manufacturer’s directions. PVDF membranes were blocked for 1 h in 5% non-fat milk in TBS-T (Bio-Rad) and incubated overnight at 4°C with anti-MBTD1 (1:500, ab170848, Abcam) and anti-GAPDH (1:3000, #2118S Cell Signaling Technology, United States) in 5% BSA in TBS-T. Blots were then probed with anti-rabbit IgG conjugated with horseradish peroxidase (1:5000, #7074, Cell Signaling Technology) in 5% BSA in TBS-T for 1 h at room temperature. Signal was detected with the Immobilon Western Chemiluminescent HRP Substrate (Millipore, MA, United States), and blot images were collected with a Bio-Rad ChemiDoc imaging system. Image Lab was used for densitometry analysis of the blot.

### Immunofluorescence

Formalin-fixed, paraffin-embedded sections (5 μm) of human menstrual endometrium were deparaffinized in xylene, rehydrated in an ethanol gradient, and then boiled in antigen retrieval citrate-buffer (Vector Laboratories Inc., CA, United States). Subsequently, the samples were blocked with 2.5% goat serum in PBS (Vector Laboratories) for 1 h at room temperature. Sections were then incubated overnight at 4°C with MBTD1 antibody (ab170848, Abcam) or normal rabbit IgG (#2729, Cell Signaling Technology) diluted 1:50 in 2.5% normal goat serum. After washing with PBS, sections were incubated with Alexa Fluor 488-conjugated secondary antibody (Life Technologies) diluted 1:500 in 2.5% normal goat serum for 1 h at room temperature. Thereafter, the sections were washed with PBS (3 × 5 min each) and mounted with ProLong Gold Antifade Mountant with DAPI (cat. no. P36962 Thermo Scientific). Images were captured using a confocal microscope (Leica DMI 4000B).

### Immunocytochemistry

HESCs were grown on a coverslip coated with poly-L-lysine (cat. no. P4832 Sigma-Aldrich) in six-well plates. After 6 days of *in vitro* decidualization as described above, cells were fixed with 4% paraformaldehyde (Alfa Aesar, United States) in PBS for 20 min at room temperature. Cells were then washed three times with PBS and permeabilized with 0.2% Triton X-100 (Sigma Aldrich, United States) in PBS for 20 min at room temperature. Next, cells were washed with PBS, blocked with 2.5% normal goat serum (Vector Laboratories) in PBS for 1 h at room temperature and incubated overnight at 4°C with anti-MBTD1 (ab170848, Abcam, 1:50 dilution) in 2.5% normal goat serum. After washing with PBS, the cells were incubated with Alexa Fluor 488-conjugated secondary antibodies (Life Technologies, 1:500 dilution) for 1 h at room temperature, washed, and mounted with ProLong Gold Antifade Mountant with DAPI (Thermo Scientific). Images were captured with a confocal microscope (Leica DMI 4000B) (Michalski et al., 2018).

### Mice and Hormone Treatments

All experimental procedures involving mice followed a protocol approved by the Washington University in St. Louis Institutional Animal Care and Use Committee (Protocol Number 20160227). CD1 wild-type mice (Charles River, Saint Louis, MO, United States) were maintained on a 12-h light:12-h dark cycle. To assess uterine progesterone responses, 6-week-old CD1 mice were bilaterally ovariectomized, allowed to rest for 2 weeks to allow the endogenous ovarian-derived steroid hormones to dissipate, and then subcutaneously injected with 100 μl of sesame oil (vehicle control) or 1 mg of progesterone (Sigma-Aldrich) in 100 μl of sesame oil. Six hours later, mice were euthanized, uterine tissues were collected, and RNA was isolated and processed for qRT-PCR.

### Statistical Analysis

A two-tailed paired Student’s *t*-test was used to analyze experiments comparing two experimental groups, and analysis of variance (ANOVA) by non-parametric alternatives was used for multiple comparisons to analyze experiments containing more than two groups. A value of *p* < 0.05 was considered significant. All data are presented as mean ± SEM. GraphPad Prism 8 software was used for all statistical analyses.

## Results

### MBTD1 Expression Is Increased in Stromal Cells of Secretory Phase Endometrium

**FIGURE 1:**
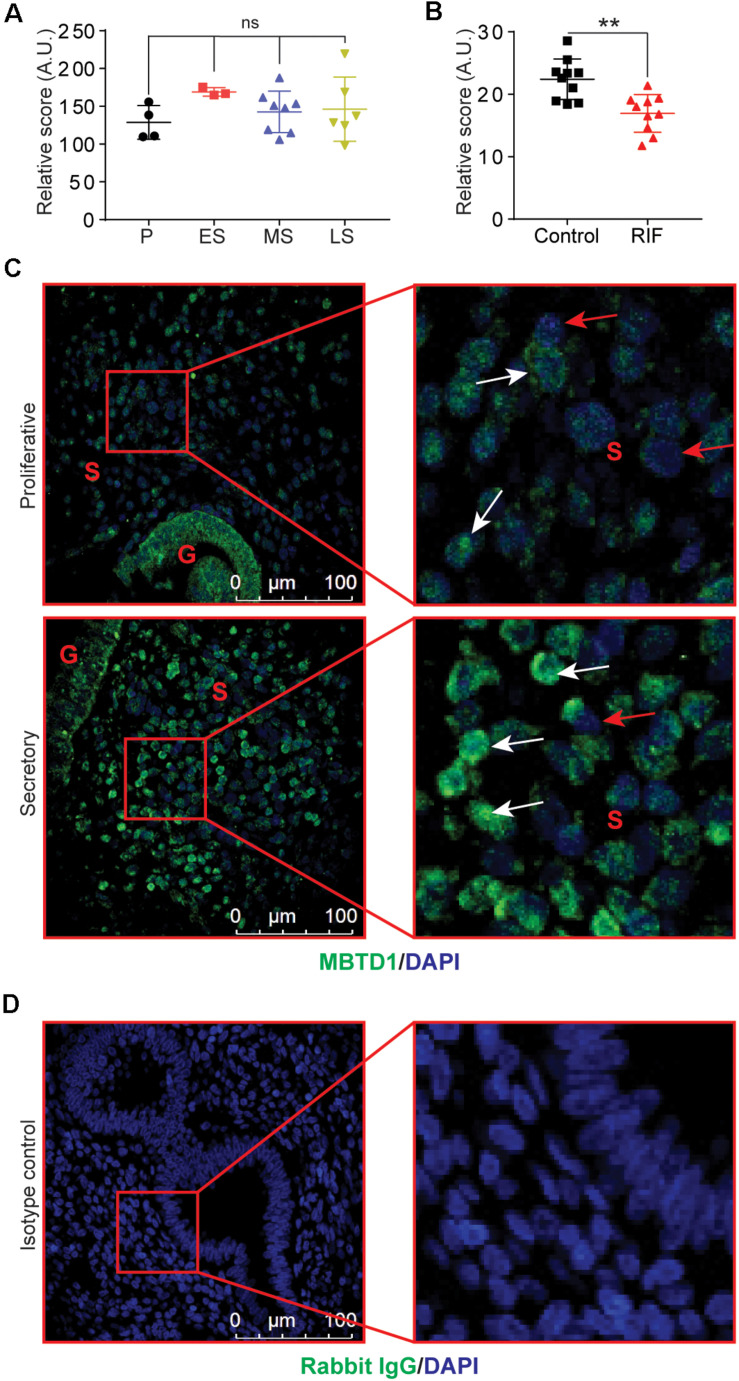
Endometrial expression of MBTD1 throughout the menstrual cycle. **(A)** *MBTD1* raw transcript scores of normal endometrium at various phases of the menstrual cycle, from microarray analysis within a publicly available GEO dataset (GSE4888). Results shown as mean ± SEM (*n* = 3–7). P, proliferative; ES, early secretory; MS, mid-secretory; and LS, late secretory. **(B)** *MBTD1* raw transcript scores in the mid-secretory phase endometrium from women with and without recurrent implantation failure (RIF) measured from publicly available GEO data set (GSE65102) in patients. Results shown as mean ± SEM (*n* = 10). **(C)** Immunolocalization of MBTD1 (green) in proliferative and secretory phase endometrium (*n* = 9 proliferative and *n* = 6 secretory) showing increased nuclear localization in stromal cells of secretory phase endometrium. White arrowheads indicate MBTD1-positive cells, and red arrowheads indicate MBTD1-negative cells. Blue stain is DAPI. G, gland; S, stroma. **(D)** Rabbit IgG was used as isotype control for staining. Scale bar: 100 μm, **P < 0.01 and ns, non-significant.

To explore the role of MBTD1 in endometrial function, we took advantage of publicly available data sets to analyze *MBTD1* expression in normal endometrium at various stages of the menstrual cycle. We found a modest increase in the *MBTD1* raw expression score during the secretory phase of the menstrual cycle as compared to the proliferative phase (Figure 1A, GSE4888) (Talbi et al., 2006). Interestingly, *MBTD1* raw expression score was significantly reduced in the endometrium from women with recurrent implantation failure compared to healthy women (Figure 1B, GSE65102) (Lucas et al., 2016). These analyses indicate that *MBTD1* expression differs in women with and without endometrial dysfunction. To determine where MBTD1 is expressed in the endometrium, we assessed MBTD1 protein immunoreactivity in the endometrium from proliferative and secretory phases (Figure 1C). We found an increased number of MBTD1 stained cells in stroma of secretory phase endometrium compared to proliferative phase endometrium (Figure 1C). Rabbit IgG was used as an isotype control to ensure the specificity of MBTD1 antibody (Figure 1D). These results suggest that MBTD1 might play a role in endometrial decidualization, and aberrant expression might be associated with uterine disorders that affect fertility such as recurrent implantation failure. Given this, in-depth functional analyses are warranted.

### *MBTD1* Promotes Decidualization in Transformed HESCs

**FIGURE 2:**
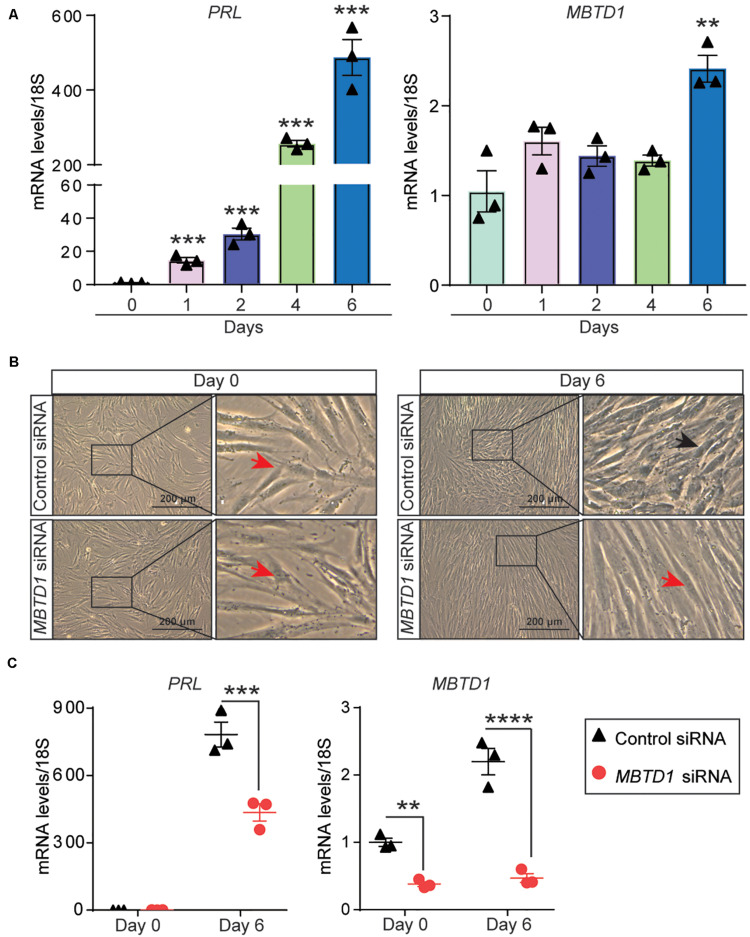
MBTD1 is required for decidualization of immortalized human endometrial stromal cells (HESCs). **(A)** Levels of *PRL* and *MBTD1* transcripts from immortalized HESCs after induction of decidualization over 6 days. **(B)** Representative images of transformed HESCs transfected with control or *MBTD1* siRNA prior to (day 0) and after 6 days of culture in decidualization (EPC, Estrogen-MPA-cAMP) conditions. Red arrowheads indicate non-decidualized cells, and black arrowheads indicate decidualized cells. Scale bar: 200 μm. **(C)** Levels of *MBTD1* and *PRL* transcripts in immortalized HESCs transfected with control or *MBTD1* siRNA after 6 days of decidualization. Results are mean ± SEM from three biological replicates; **P < 0.01, ***P < 0.001, and ****P < 0.0001.

Given that endometrial decidualization occurs during the secretory phase of the menstrual cycle, and MBTD1 expression is elevated in secretory phase endometrium, we questioned whether MBTD1 has a role in endometrial decidualization. To test this, we first used T-HESCs as a model for *in vitro* decidualization. When exposed to decidualization conditions for 6 days, T-HESCs exhibited a progressive increase in the expression of the decidual marker prolactin (*PRL*) (Szwarc et al., 2018a; Figure 2A). By day 6 of decidualization, T-HESCs also expressed over two-fold more *MBTD1* transcript than at day 0 (Figure 2A). Next, we transfected the transformed HESCs with either control siRNA or siRNA targeting *MBTD1*. As expected, control siRNA-transfected cells transformed from fibroblastic to cobblestone-like epithelioid cell morphology (Figure 2B) and showed increased expression of *PRL* and *MBTD1* by day 6 of decidualization (Figure 2C). In contrast, cells transfected with *MBTD1* siRNA failed to undergo this morphological change (Figure 2B) and did not have elevated expression of *PRL* or *MBTD1* (Figure 2C). These results indicate that MBTD1 promotes decidualization in transformed HESCs.

### MBTD1 Is Induced During Decidualization in Primary HESCs

**FIGURE 3:**
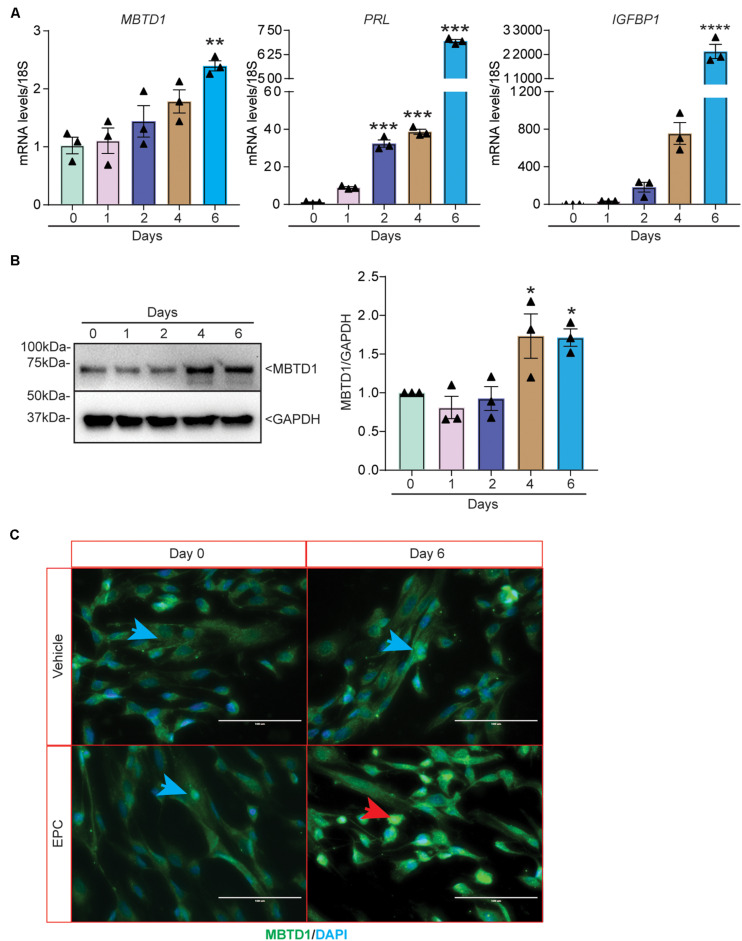
MBTD1 expression is induced during *in vitro* decidualization of primary human endometrial stromal cells (HESCs). **(A)** Levels of *MBTD1*, *PRL*, and *IGFBP1* transcripts from HESCs induced to decidualize for 6 days. **(B)** Western blot and densitometric analysis of MBTD1 protein levels from HESCs cultured in decidualization media for 6 days. GAPDH was used as a loading control. **(C)** Immunofluorescent detection of MBTD1 (green) in HESCs treated with vehicle or decidualization media (EPC, Estrogen-MPA-cAMP) prior to (day 0) and after decidualization (day 6). Prominent MBTD1 nuclear staining was apparent in decidualized HESCs. Blue stain is DAPI. Red arrowhead indicates decidualized cells and blue arrowheads indicate non-decidualized cells. Scale bar: 100 μm. Results are shown as the mean ± SEM from three biological replicates from a representative experiment (experiment repeated three times, *n* = 3); *P < 0.05, **P < 0.01, ***P < 0.001, and ****P < 0.0001.

We next sought to determine the role of MBTD1 in primary HESC decidualization. Similar to our findings in transformed HESCs, *MBTD1* transcripts increased by 6 days of decidualization in primary HESCs (Figure 3A). Induction of the decidual markers *PRL* (Szwarc et al., 2018a) and insulin-like growth factor-binding protein-1 (*IGFBP1*) (Szwarc et al., 2018b) confirmed that the HESCs effectively decidualized (Figure 3A). MBTD1 protein expression also increased during decidualization, as evidenced both by Western blot (Figure 3B) and by immunofluorescence (Figure 3C). These data are in line with the elevated expression of stromal-derived MBTD1 in secretory phase endometrium (Figure 1C). As expected, MBTD1 protein was predominantly nuclear in both non-decidualized and decidualized primary HESCs (Figure 3C). The presence of MBTD1 in the non-decidualized cells is consistent with its basal level expression in the proliferative phase endometrium (Figure 1C).

### MBTD1 Is Essential for Decidualization of Primary HESCs

**FIGURE 4:**
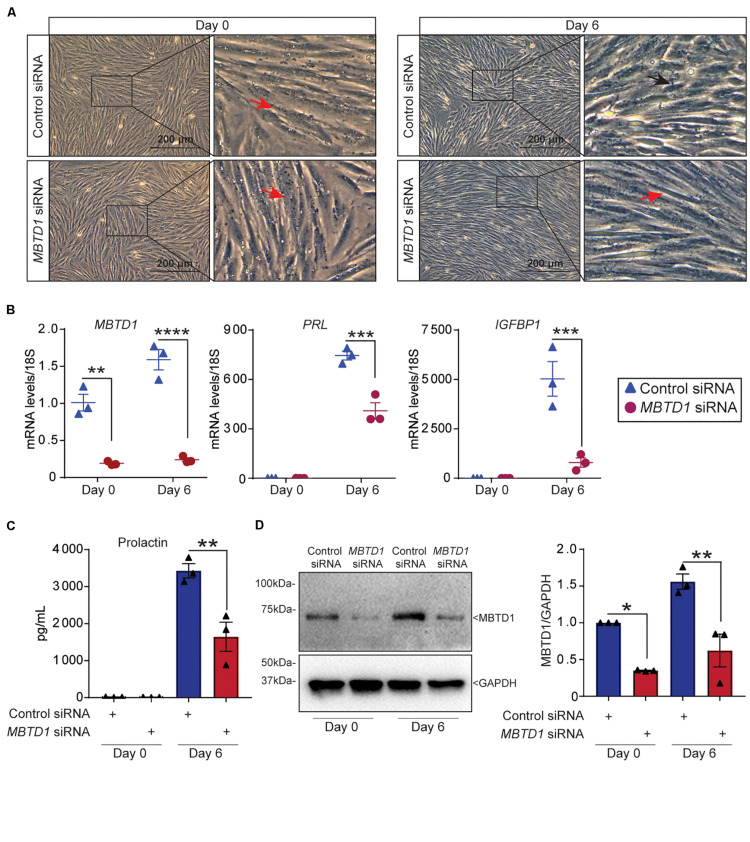
MBTD1 is essential for primary human endometrial stromal cell decidualization. **(A)** Morphology of human endometrial stromal cells (HESCs) transfected with control or *MBTD1* siRNA at basal (day 0) or after 6 days of culture in decidualization conditions. Red arrowheads indicate non-decidualized cells and black arrowhead indicates decidualized cells. Scale bar: 200 μm. **(B)** Levels of *MBTD1, IGFBP1*, and *PRL* transcripts in HESCs transfected with control or *MBTD1* siRNAs at basal (day 0) or 6 days of decidualization. **(C)** Levels of secreted Prolactin into the media were measured using ELISA-based assay in HESCs transfected with control or *MBTD1* siRNAs at day 0 or 6 days after EPC treatment. **(D)** Western blot and densitometric analysis of MBTD1 protein levels from HESCs transfected with control or *MBTD1* siRNA. Representative data from three replicates from one patient sample are shown as mean ± SEM. The experiment was repeated three times, *n* = 3; *P < 0.05, **P < 0.01, ***P < 0.001, and ****P < 0.0001.

To determine whether MBTD1 was required for primary HESC decidualization, we transfected HESCs with control or *MBTD1*-targeting siRNAs and exposed the cells to decidualization conditions. HESCs that received control siRNA changed morphology (Figure 4A) and had increased expression of *MBTD1*, *IGFBP1*, and *PRL* (Figure 4B) by day 6. In contrast, HESCs that received *MBTD1*-targeting siRNA did not show a dramatic morphological change over 6 days (Figure 4A) and expressed significantly less *MBTD1*, *PRL*, and *IGFBP1* than control cells (Figure 4B). Further, quantification of secreted Prolactin (PRL) from culture media revealed the significant decrease at day 6 in HESCs that received *MBTD1*-targeting siRNA (Figure 4C). The knockdown efficiency of MBTD1 at protein level was confirmed by immunoblotting (Figure 4D). As expected, siRNA against *MBTD1* effectively downregulated MBTD1 protein levels at both basal (day 0) and induced (day 6) time points in decidualizing stromal cells. Together, these results indicate that *MBTD1* is required for complete primary HESC decidualization.

### MBTD1 Controls Cell Proliferation and Cell Cycle Progression of Decidualizing HESCs

**FIGURE 5:**
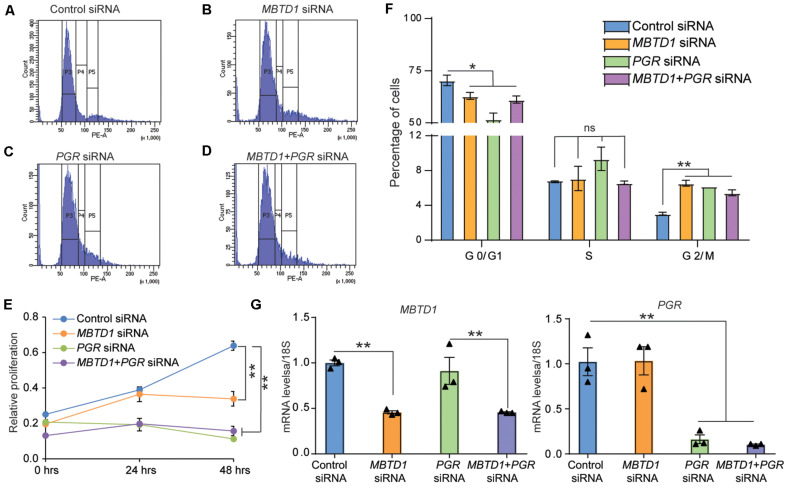
MBTD1 controls human endometrial stromal cell proliferation and cell cycle progression. **(A–D)** Human endometrial stromal cells (HESCs) transfected with control **(A)**, *MBTD1* **(B)**, *PGR* **(C)**, and *PGR* + *MBTD1* siRNAs **(D)**. After 48 h of transfection, cells were treated in decidualization media for 48 h and subjected to flow cytometry analysis. Histograms depict the percentages of cells in each phase of the cell cycle. **(E)** Graph depicting the distribution of cells in indicated phases of the cell cycle. **(F)** MTT cell proliferation assay of HESCs transfected with control siRNA or *MBTD1* siRNA or *PGR* siRNA or *MBTD1* + *PGR* siRNAs at indicated time points. **(G)** Transcript levels of *MBTD1* (left panel) and *PGR* (right panel) from HESCs after 48 h of transfection with indicated siRNAs. Representative data from one patient sample and the experiment repeated three times with independent HES cell lines. Results represent the mean ± SEM (*n* = 3). *P < 0.05, **P < 0.01, ns, non-significant.

Given that stromal cell decidualization is regulated by many factors that control cell cycle progression, we next examined the effect of MBTD1 on cell cycle progression. To test this, we transiently transfected HESCs with control siRNAs or *MBTD1* siRNA or *PGR* siRNA or *MBTD1* and *PGR* siRNAs together, cultured the cells for 48 h in EPC media, and then subjected them to flow cytometry. Knockdown of either *MBTD1* or *PGR* resulted in an increase in G2/M arrested cells, with a concomitant reduction in G0/G1 cells (Figures 5A–E). Interestingly, we found no synergistic effect of MBTD1 and PGR in mediating HESC proliferation. The effect of PGR knockdown on G0/G1and G2/M is consistent with published work (Logan et al., 2012) that found MPA augments G2/M cell cycle arrest of HESCs during decidualization. Since alterations in cell cycle progression impacts cell survival, we next examined the effect of MBTD1 on stromal cell proliferation. Consistent with altered cell cycle progression, we found that knockdown of either *MBTD1* or *PGR* or both together resulted in a decreased HESC proliferation (Figure 5F). Additionally, we confirmed the effective knockdown of *MBTD1* and *PGR* (Figure 5G) with the respective siRNAs. These results suggest that MBTD1 controls cell proliferation and cell cycle progression of decidualizing HESCs.

### MBTD1 Regulates Expression of Progesterone Receptor Target Genes

**FIGURE 6:**
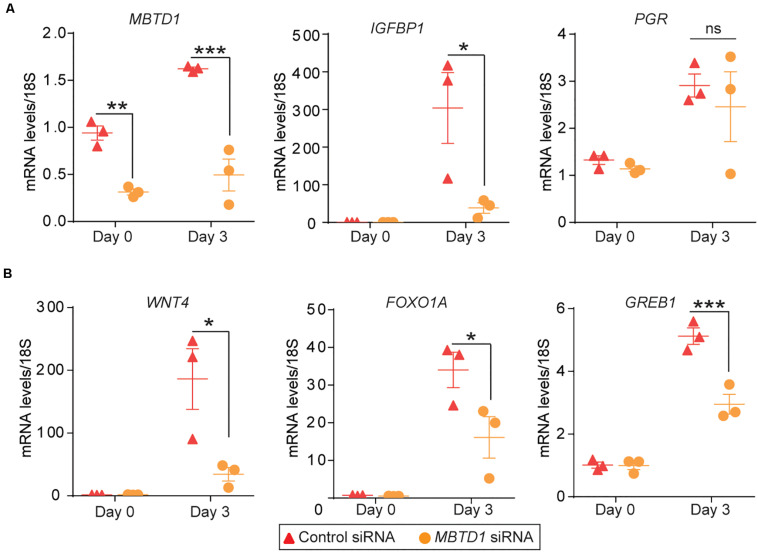
MBTD1 is required for induction of key decidual marker genes in decidualizing primary HESCs. **(A)** Transcript levels of *MBTD1*, *IGFBP1*, and *PGR* in HESCs transfected with control or *MBTD1* siRNAs and induced to decidualize for 3 days. **(B)** *WNT4, FOXO1A*, and *GREB1* expression in HESCs transfected with control or *MBTD1* siRNAs and induced to decidualize for 3 days. Representative data from three replicates from one patient sample are shown as mean ± SEM. The experiment repeated three times, *n* = 3; *P < 0.05, **P < 0.01, ***P < 0.001, ns, non-significant.

To define the molecular mechanism by which *MBTD1* promotes decidualization, we evaluated the effect of *MBTD1* knockdown on expression of progesterone receptor (PGR) target genes *WNT4*, *FOXO1A*, and *GREB1*, which together promote HESC decidualization (Kommagani et al., 2016; Camden et al., 2017). In primary HESCs, transfected with control siRNA, expression of *IGFBP1*, *PGR*, *WNT1*, *FOXO1A*, and *GREB1* all increased after 3 days in decidualization conditions (Figures 6A,B). Primary HESCs transfected with *MBTD1* siRNA had significantly less *IGFBP1*, *WNT4, FOXO1A*, and *GREB1* transcripts after 3 days than did cells transfected with control siRNA (Figures 6A,B). However, expression of *PGR* was not affected by *MBTD1* knockdown in decidualizing HESCs (Figure 6A). These results indicate that MBTD1 modulates PGR-mediated gene expression but not *PGR* expression during endometrial decidualization.

### MBTD1 Is Not Responsive to Progesterone in Human or Mouse Endometrium

**FIGURE 7:**
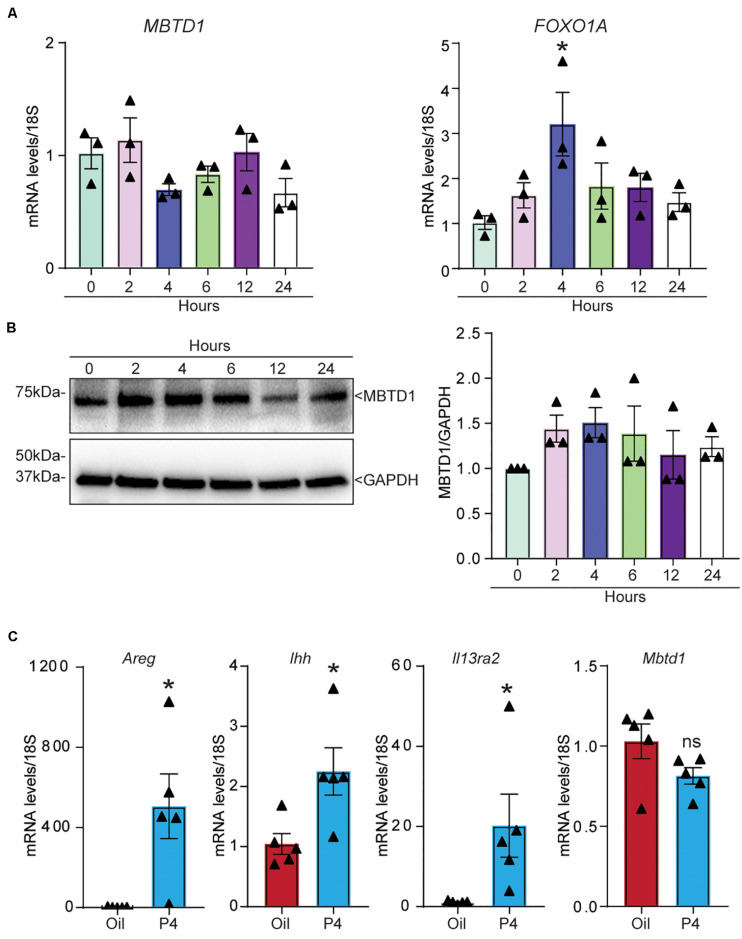
MBTD1 is not a progesterone-responsive gene in human or mouse endometrium. **(A,B)** Relative amounts of *MBTD1* and *FOXO1A* mRNA **(A)** and MBTD1 protein with densitometric analysis **(B)** in human endometrial stromal cells (HESCs) treated with 1 μM MPA over 24 h. Representative data from three replicates from one patient sample are shown as mean ± SEM. GAPDH was used as a loading control. **(C)** The relative transcript levels of *Areg* (amphiregulin), *Ihh* (Indian hedgehog), and *Il13ra2* (interleukin 13 receptor subunit alpha 2) and *Mbtd1* in uteri from ovariectomized mice treated with vehicle (oil) or 1 mg progesterone (P4) for 6 h. Results represent the mean ± SEM; *n* = 5 mice/group. *P < 0.05, and ns, non-significant.

Finally, we wondered whether MBTD1 expression was regulated by progesterone. The levels of both *MBTD1* transcript (Figure 7A) and MBTD1 protein (Figure 7B) remained unchanged in primary HESCs over 24 h of treatment with MPA. Induction of the well-established PGR target *FOXO1A* at 4 h post-MPA treatment confirmed that the cells responded to MPA (Figure 7A). To determine the physiological relevance of this finding, we measured *Mbtd1* gene expression in uteri from ovariectomized mice treated with progesterone (P4) for 6 h. Although this treatment increased uterine expression of the well-established progesterone targets amphiregulin (*Areg*), Indian hedgehog (*Ihh*), and interleukin 13 receptor subunit alpha 2 (*Il13ra2*) (Figure 7C; Kommagani et al., 2016; Rubel et al., 2016), progesterone treatment did not increase expression of *Mbtd1* (Figure 7C). Together, these data indicate that *MBTD1* is not a direct target of progesterone in human or murine endometrium.

## Discussion

In humans, endometrial stromal cell decidualization begins spontaneously during the mid-secretory phase of the menstrual cycle, even if a conceptus is not present (Zhang et al., 2013). Successful endometrial stromal cell decidualization is one of the critical events during early pregnancy and is required for establishment of successful pregnancy. Increasing evidence suggests that impaired decidualization is the leading cause in a variety of fertility-associated conditions including early miscarriages, recurrent implantation failures (Salamonsen et al., 2009; Wang and Yu, 2018; Messaoudi et al., 2019), recurrent pregnancy loss, and preeclampsia (Brosens et al., 2002; Salker et al., 2010). Specifically, impaired decidualization contributes to ∼30% of failed pregnancies and is a significant cause for recurrent implantation failures in *in vitro* fertilization. Therefore, even with high-quality embryo transfers, implantation failures occur due to endometrial dysfunction.

Here, we provided five lines of evidence indicating that MBTD1 participates in HESC decidualization by promoting progesterone signaling. First, we showed that MBTD1 is predominantly localized in the HESCs of secretory phase endometrium. Second, MBTD1 expression was induced during *in vitro* decidualization of both immortalized and primary HESCs. Third, siRNA-mediated *MBTD1* knockdown impaired *in vitro* decidualization of both immortalized and primary HESCs. Fourth, MBTD1 controls the HESC proliferation and cell cycle progression during decidualization. Fifth, we showed that MBTD1 mediates transcription of the PR targets *WNT4*, *FOXOA1*, and *GREB1*. However, MBTD1 expression was not regulated by progesterone.

We found that MBTD1 controls cell cycle progression during stromal cell decidualization, which is consistent with its established role in cell cycle regulation. For example, Luo et al. (2013) showed that depletion of MBTD1 in oocytes activated the cell cycle checkpoint protein Chk1 and led to cell cycle arrest. Further, regulation of HESC proliferation by MBTD1 is in line with published work that indicated that MBTD1 controls growth of multiple cancer cells (de Rooij et al., 2016; Hoang et al., 2018; Yamamoto et al., 2018; Fu et al., 2019; Plesa and Sujobert, 2019; Wu et al., 2019). Thus, based on our findings and published work, we posit that MBTD1 functions in HESC decidualization through multiple cellular mechanisms. First, MBTD1 could control the stromal cell proliferation that precedes differentiation and cell cycle progression of stromal cells to differentiate into decidual cells. Second, MBTD1 could participate in protecting HESCs from DNA damage. Recent reports indicate that DNA damage increases during decidualization (Lei et al., 2012). Specifically, significantly higher levels of H2AX phosphorylation, a marker of DNA damage, were observed at implantation sites compared to inter-implantation sites in rodents (Lei et al., 2012; Yang et al., 2018). Additionally, expression of the DNA damage repair and DNA binding proteins is induced during the mid- to late-secretory phase of the menstrual cycle and is expressed in decidualizing ESCs *in vitro* (Kajihara et al., 2006; Labied et al., 2006). Furthermore, Luo et al. showed that depletion of MBTD1 in mouse oocytes led to downregulation of the DNA damage repair checkpoint protein 53BP1 and increased formation of γH2AX foci (Luo et al., 2013). Future work should investigate the ability of MBTD1 to contribute to genomic stability during decidualization.

Given the results presented here and the established roles of MBTD1, we posit that MBTD1 enables progesterone-dependent differentiation of HESCs into decidual cells. Further studies investigating the role of MBTD1 *in vivo* will aid in developing new diagnostics and therapeutics for endometrial pathologies that affect fertility such as recurrent pregnancy loss.

## Data Availability Statement

The datasets generated for this study are available on request to the corresponding author.

## Ethics Statement

The studies involving human participants were reviewed and approved by Washington University in St. Louis Institutional Review Board (ID #201612127). The patients/participants provided their written informed consent to participate in this study. The animal study was reviewed and approved by Washington University in St. Louis Institutional Animal Care and Use Committee (Protocol Number 20160227).

## Author Contributions

SC and RK designed the experiments, conducted the studies, analyzed the data, and wrote and edited the manuscript. VM and GK assisted in select experiments and manuscript writing and editing. RK conceived the project, supervised the work, and provided resources. EJ assisted with obtaining donor biopsies. All authors reviewed and approved the final version of the manuscript.

## Conflict of Interest

The authors declare that the research was conducted in the absence of any commercial or financial relationships that could be construed as a potential conflict of interest.

## References

[B1] Bian F. Gao F. Kartashov A. V. Jegga A. G. Barski A. Das S. K. (2016). Polycomb repressive complex 1 controls uterine decidualization. *Sci. Rep.* 6:26061. 10.1038/srep26061 PMC4867636 27181215

[B2] Brosens J. J. Pijnenborg R. Brosens I. A. (2002). The myometrial junctional zone spiral arteries in normal and abnormal pregnancies: a review of the literature. *Am. J. Obstet. Gynecol.* 187 1416–1423. 10.1067/mob.2002.127305 12439541

[B3] Camden A. J. Szwarc M. M. Chadchan S. B. DeMayo F. J. O’Malley B. W. Lydon J. P. (2017). Growth regulation by estrogen in breast cancer 1 (GREB1) is a novel progesterone-responsive gene required for human endometrial stromal decidualization. *Mol. Hum. Reprod.* 23 646–653. 10.1093/molehr/gax045 28911214 PMC5909854

[B4] de Rooij J. D. van den Heuvel-Eibrink M. M. Kollen W. J. Sonneveld E. Kaspers G. J. Beverloo H. B. (2016). Recurrent translocation t(10;17)(p15;q21) in minimally differentiated acute myeloid leukemia results in ZMYND11/MBTD1 fusion. *Genes Chromosomes Cancer* 55 237–241. 26608508 10.1002/gcc.22326

[B5] Dewaele B. Przybyl J. Quattrone A. Finalet Ferreiro J. Vanspauwen V. Geerdens E. (2014). Identification of a novel, recurrent MBTD1-CXorf67 fusion in low-grade endometrial stromal sarcoma. *Int. J. Cancer* 134 1112–1122. 10.1002/ijc.28440 23959973

[B6] Fu D. Lu C. Qu X. Li P. Chen K. Shan L. (2019). LncRNA TTN-AS1 regulates osteosarcoma cell apoptosis and drug resistance via the miR-134-5p/MBTD1 axis. *Aging* 11 8374–8385. 10.18632/aging.102325 31600142 PMC6814585

[B7] Hantak A. M. Bagchi I. C. Bagchi M. K. (2014). Role of uterine stromal-epithelial crosstalk in embryo implantation. *Int. J. Dev. Biol.* 58 139–146. 10.1387/ijdb.130348mb 25023679 PMC4768910

[B8] Hoang L. Chiang S. Lee C. H. (2018). Endometrial stromal sarcomas and related neoplasms: new developments and diagnostic considerations. *Pathology* 50 162–177. 10.1016/j.pathol.2017.11.086 29275929

[B9] Jacquet K. Fradet-Turcotte A. Avvakumov N. Lambert J. P. Roques C. Pandita R. K. (2016). The TIP60 complex regulates bivalent chromatin recognition by 53BP1 through direct H4K20me binding and H2AK15 acetylation. *Mol. Cell.* 62 409–421. 10.1016/j.molcel.2016.03.031 27153538 PMC4887106

[B10] Kajihara T. Jones M. Fusi L. Takano M. Feroze-Zaidi F. Pirianov G. (2006). Differential expression of FOXO1 and FOXO3a confers resistance to oxidative cell death upon endometrial decidualization. *Mol. Endocrinol.* 20 2444–2455. 10.1210/me.2006-0118 16709600

[B11] Kommagani R. Szwarc M. M. Kovanci E. Gibbons W. E. Putluri N. Maity S. (2013). Acceleration of the glycolytic flux by steroid receptor coactivator-2 is essential for endometrial decidualization. *PLoS Genet.* 9:e1003900. 10.1371/journal.pgen.1003900 24204309 PMC3812085

[B12] Kommagani R. Szwarc M. M. Vasquez Y. M. Peavey M. C. Mazur E. C. Gibbons W. E. (2016). The promyelocytic leukemia zinc finger transcription factor is critical for human endometrial stromal cell decidualization. *PLoS Genet.* 12:e1005937. 10.1371/journal.pgen.1005937 27035670 PMC4817989

[B13] Koot Y. E. Teklenburg G. Salker M. S. Brosens J. J. Macklon N. S. (2012). Molecular aspects of implantation failure. *Biochim. Biophys. Acta* 1822 1943–1950. 22683339 10.1016/j.bbadis.2012.05.017

[B14] Labied S. Kajihara T. Madureira P. A. Fusi L. Jones M. C. Higham J. M. (2006). Progestins regulate the expression and activity of the forkhead transcription factor FOXO1 in differentiating human endometrium. *Mol. Endocrinol.* 20 35–44. 10.1210/me.2005-0275 16123151

[B15] Lei W. Feng X. H. Deng W. B. Ni H. Zhang Z. R. Jia B. (2012). Progesterone and DNA damage encourage uterine cell proliferation and decidualization through up-regulating ribonucleotide reductase 2 expression during early pregnancy in mice. *J. Biol. Chem.* 287 15174–15192. 10.1074/jbc.m111.308023 22403396 PMC3346129

[B16] Levine S. S. Weiss A. Erdjument-Bromage H. Shao Z. Tempst P. Kingston R. E. (2002). The core of the polycomb repressive complex is compositionally and functionally conserved in flies and humans. *Mol. Cell. Biol.* 22 6070–6078. 10.1128/mcb.22.17.6070-6078.2002 12167701 PMC134016

[B17] Liu H. Huang X. Mor G. Liao A. (2019). Epigenetic modifications working in the decidualization and endometrial receptivity. *Cell Mol. Life Sci.* 77 2091–2101. 10.1007/s00018-019-03395-9 31813015 PMC11105058

[B18] Logan P. C. Steiner M. Ponnampalam A. P. Mitchell M. D. (2012). Cell cycle regulation of human endometrial stromal cells during decidualization. *Reprod. Sci.* 19 883–894. 10.1177/1933719112438447 22534328

[B19] Lonard D. M. O’Malley B. W. (2012). Nuclear receptor coregulators: modulators of pathology and therapeutic targets. *Nat. Rev. Endocrinol.* 8 598–604. 10.1038/nrendo.2012.100 22733267 PMC3564250

[B20] Lucas E. S. Dyer N. P. Murakami K. Lee Y. H. Chan Y. W. Grimaldi G. (2016). Loss of endometrial plasticity in recurrent pregnancy loss. *Stem Cells* 34 346–356. 26418742 10.1002/stem.2222

[B21] Luo Y. B. Ma J. Y. Zhang Q. H. Lin F. Wang Z. W. Huang L. (2013). MBTD1 is associated with Pr-Set7 to stabilize H4K20me1 in mouse oocyte meiotic maturation. *Cell Cycle* 12 1142–1150. 10.4161/cc.24216 23475131 PMC3646870

[B22] Messaoudi S. El Kasmi I. Bourdiec A. Crespo K. Bissonnette L. Le Saint C. (2019). 15 years of transcriptomic analysis on endometrial receptivity: what have we learnt? *Fertil. Res. Pract.* 5:9. 10.1186/s40738-019-0059-7 PMC6681490 31396393

[B23] Michalski S. A. Chadchan S. B. Jungheim E. S. Kommagani R. (2018). Isolation of human endometrial stromal cells for in vitro decidualization. *J. Vis. Exp.* 139:57684. 10.3791/57684 PMC6235076 30222162

[B24] Mills A. A. (2010). Throwing the cancer switch: reciprocal roles of polycomb and trithorax proteins. *Nat. Rev. Cancer* 10 669–682. 10.1038/nrc2931 20865010 PMC4068012

[B25] Plesa A. Sujobert P. (2019). Cannibalistic acute myeloid leukemia with ZMYND11-MBTD1 fusion. *Blood* 133:1789. 10.1182/blood-2019-01-898619 31000516

[B26] Rubel C. A. Jeong J. W. Tsai S. Y. Lydon J. P. Demayo F. J. (2010). Epithelial-stromal interaction and progesterone receptors in the mouse uterus. *Semin. Reprod. Med.* 28 27–35. 20104426 10.1055/s-0029-1242990

[B27] Rubel C. A. Wu S. P. Lin L. Wang T. Lanz R. B. Li X. (2016). A Gata2-dependent transcription network regulates uterine progesterone responsiveness and endometrial function. *Cell. Rep.* 17 1414–1425. 10.1016/j.celrep.2016.09.093 27783953 PMC5084852

[B28] Salamonsen L. A. Nie G. Hannan N. J. Dimitriadis E. (2009). Society for reproductive biology founders’ Lecture 2009. Preparing fertile soil: the importance of endometrial receptivity. *Reprod. Fertil. Dev.* 21 923–934. 19698296 10.1071/RD09145

[B29] Salker M. Teklenburg G. Molokhia M. Lavery S. Trew G. Aojanepong T. (2010). Natural selection of human embryos: impaired decidualization of endometrium disables embryo-maternal interactions and causes recurrent pregnancy loss. *PLoS One* 5:e10287. 10.1371/journal.pone.0010287 20422017 PMC2858209

[B30] Szwarc M. M. Hai L. Gibbons W. E. Peavey M. C. White L. D. Mo Q. (2018a). Human endometrial stromal cell decidualization requires transcriptional reprogramming by PLZF. *Biol. Reprod.* 98 15–27. 10.1093/biolre/iox161 29186366 PMC5819842

[B31] Szwarc M. M. Hai L. Gibbons W. E. White L. D. Mo Q. Kommagani R. (2018b). Retinoid signaling controlled by SRC-2 in decidualization revealed by transcriptomics. *Reproduction* 156 387–395. 10.1530/rep-18-0282 30325183 PMC6208442

[B32] Talbi S. Hamilton A. E. Vo K. C. Tulac S. Overgaard M. T. Dosiou C. (2006). Molecular phenotyping of human endometrium distinguishes menstrual cycle phases and underlying biological processes in normo-ovulatory women. *Endocrinology* 147 1097–1121. 10.1210/en.2005-1076 16306079

[B33] Teklenburg G. Salker M. Heijnen C. Macklon N. S. Brosens J. J. (2010). The molecular basis of recurrent pregnancy loss: impaired natural embryo selection. *Mol. Hum. Reprod.* 16 886–895. 10.1093/molehr/gaq079 20847090

[B34] Wang X. Wu S. P. DeMayo F. J. (2017). Hormone dependent uterine epithelial-stromal communication for pregnancy support. *Placenta* 60(Suppl. 1), S20–S26. 28716426 10.1016/j.placenta.2017.07.003 PMC5743625

[B35] Wang X. Yu Q. (2018). An update on the progress of transcriptomic profiles of human endometrial receptivity. *Biol. Reprod.* 98 440–448. 10.1093/biolre/ioy018 29365037

[B36] Wu W. Bai S. Zhu D. Li K. Dong W. He W. (2019). Overexpression of malignant brain tumor domain containing protein 1 predicts a poor prognosis of prostate cancer. *Oncol. Lett.* 17 4640–4646. 30944653 10.3892/ol.2019.10109 PMC6444442

[B37] Yamamoto K. Yakushijin K. Ichikawa H. Kakiuchi S. Kawamoto S. Matsumoto H. (2018). Expression of a novel ZMYND11/MBTD1 fusion transcript in CD7(+)CD56(+) acute myeloid leukemia with t(10;17)(p15;q21). *Leuk. Lymphoma* 59 2706–2710. 10.1080/10428194.2018.1464157 29911449

[B38] Yang J. Zhang Y. Tong J. Lv H. Zhang C. Chen Z. J. (2018). Dysfunction of DNA damage-inducible transcript 4 in the decidua is relevant to the pathogenesis of preeclampsia. *Biol. Reprod.* 98 821–833. 10.1093/biolre/ioy038 29447340

[B39] Zhang S. Lin H. Kong S. Wang S. Wang H. Wang H. (2013). Physiological and molecular determinants of embryo implantation. *Mol. Aspects Med.* 34 939–980. 10.1016/j.mam.2012.12.011 23290997 PMC4278353

