# Supplementary material for: A Role for Malignant Brain Tumor Domain-Containing Protein 1 in Human Endometrial Stromal Cell Decidualization
Source: Front Cell Dev Biol. 2020 Aug 11;8:745. doi: 10.3389/fcell.2020.00745 (PMC7432280; doi:10.3389/fcell.2020.00745)
Supplement: Supplementary file 1 [file Data_Sheet_1.pdf]

Supplementary Material for

A Role for Malignant Brain Tumor Domain-containing protein 1 in Human Endometrial Stromal Cell Decidualization.

Sangappa B. Chadchan, Vineet K. Maurya, Gwendalyn L. Krekeler, Emily S. Jungheim, and Ramakrishna Kommagani*

This PDF file includes

Supplementary Figures 1-3.

Supplementary Table 1.

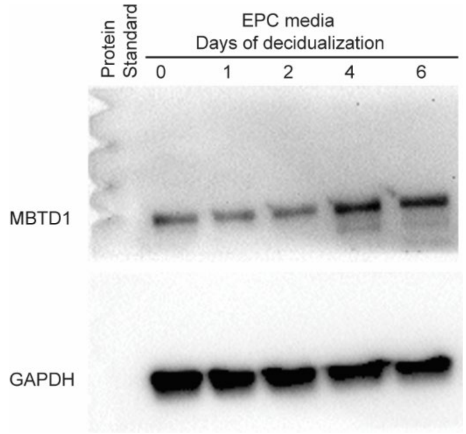

Supplementary Figure 1: MBTD1 level in human endometrial stromal cells during decidualization.

Original Western blot of MBTD1 and GAPDH as shown in Figure 3B.

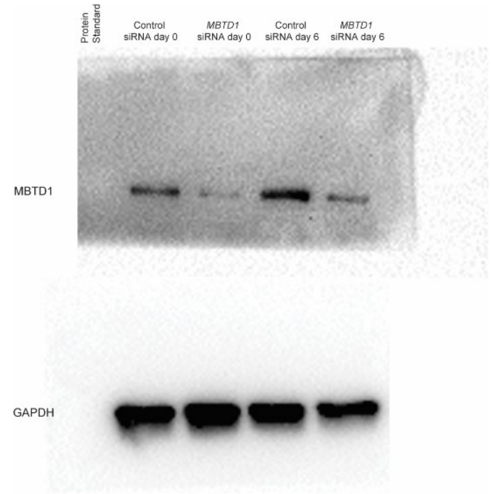

Supplementary Figure 2: MBTD1 knockdown confirmation.

Original Western blot of MBTD1 and GAPDH as shown in Figure 4D.

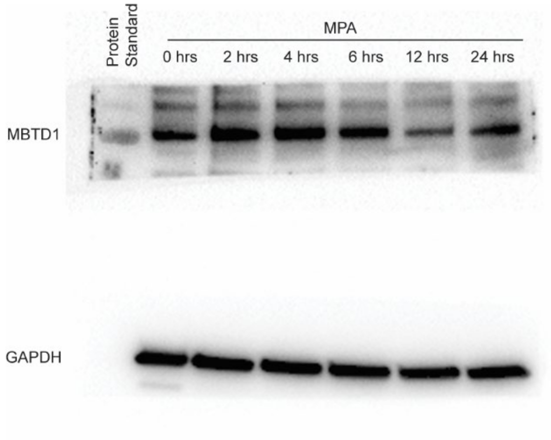

Supplementary Figure 3: Level of MBTD1 in human endometrial stromal cells during MPA treatment.

Original Western blot of MBTD1 and GAPDH as shown in Figure 7B.

Supplementary Table 1. List of TaqMan probes

Gene name	Species	Application, Chemistry	Company	Cat. No.
MBTD1	Human	qPCR, Taqman	ABI	Hs00908844_m1
PRL	Human	qPCR, Taqman	ABI	Hs00168730_m1
IGFBP1	Human	qPCR, Taqman	ABI	Hs00236877_m1
PGR	Human	qPCR, Taqman	ABI	Hs01556702_m1
WNT4	Human	qPCR, Taqman	ABI	Hs01573505_m1
GREB1	Human	qPCR, Taqman	ABI	Hs00536409_m1
FOXO1A	Human	qPCR, Taqman	ABI	Hs00231106_m1
Mbtd1	Mouse	qPCR, Taqman	ABI	Mm00506113_m1
Areg	Mouse	qPCR, Taqman	ABI	Mm01354339_m1
Ihh	Mouse	qPCR, Taqman	ABI	Mm00439613_m1
Il13ra2	Mouse	qPCR, Taqman	ABI	Mm00515166_m1
18S	Human<br>Mouse	qPCR, Taqman	ABI	4318839

ABI-applied biosystems.
